# Facet Tropism and Orientation: Risk Factors for Degenerative Lumbar Spinal Stenosis

**DOI:** 10.1155/2020/2453503

**Published:** 2020-06-29

**Authors:** Janan Abbas, Natan Peled, Israel Hershkovitz, Kamal Hamoud

**Affiliations:** ^1^Department of Anatomy and Anthropology, Sackler Faculty of Medicine, Tel Aviv University, Tel Aviv 6997801, Israel; ^2^Department of Physical Therapy, Zefat Academic College, Zefat, 13206, Israel; ^3^Department of Radiology, Carmel Medical Center, Haifa 3436212, Israel; ^4^Azrieli Faculty of Medicine, Bar-Ilan University, Safed 1311502, Israel; ^5^Department of Orthopaedic Surgery, The Baruch Padeh Poriya Medical Center, Tiberias 1520800, Israel

## Abstract

The aim of this study is to establish whether facet tropism (FT) and orientation (FO) are associated with degenerative lumbar spinal stenosis (DLSS). A retrospective computerized tomography (CT) study including 274 individuals was divided into two groups: control (82 males and 81 females) and stenosis (59 males and 52 females). All participants have undergone high-resolution CT scan of the lumbar spine in the same position. FT and FO were measured at L1-2 to L5-S1. Significant sagittal FO was noted in the stenosis males (L2-3 to L4-5) and females (L2-3 to L5-S1) compared to the controls. The prevalence of FT was remarkably greater in the stenosis males (L4-5, L5-S1) and females (L3-4, L5-S1) compared to their counterparts in the control group. Our results also showed that FT (L3-4 to L5-S1) increases approximately 2.9 times the likelihood for DLSS development. This study indicates that FO and FT in the lower lumbar spine are significantly associated with DLSS.

## 1. Introduction

The facet joint or zygapophyseal joint is a synovial joint that plays an important role in balancing the spine segment [1] and controls the spinal kinematic function [2, 3]. Lumbar facet joints are sagittally oriented compared with the lower thoracic region, and their orientation varies between the lumbar levels [4, 5]. Facet tropism (FT) is defined as a difference between the angle of orientation of the right and left facet joints [6]. Additionally, computed tomography (CT) scan is considered the most accurate modality for evaluation of the facet joint [7].

Some authors have correlated the sagittal orientation of the facet joint with degenerative spondylolisthesis (DS) [6, 8–13] and disc disease [14]. Previous studies have also reported that FT is associated with spine degeneration such as facet joint arthrosis [15, 16], degenerative scoliosis [17, 18], disc herniation [18–22], and ligamentum flavum thickening [23, 24]. Others, however, have disputed some of these associations [6, 25, 26]. Although one study [27] has recently reported that DLSS population manifested significantly smaller facet orientations compared to control yet, no study has investigated, to date, whether facet asymmetry can affect DLSS.

The aim of this study was to establish whether facet structures such as orientation and tropism along all lumbar levels are associated with DLSS population.

## 2. Materials and Methods

### 2.1. Study Groups

This is a cross-sectional retrospective study with two groups of individuals. The first one is the control group with 163 subjects (sex ratio: 82M/81F) without spinal stenosis-related symptoms (according to their medical records and interviews) who were referred to the Department of Radiology, Carmel Medical Center, Haifa, Israel, for abdominal CT scans due to abdominal problems. The second included 111 individuals (sex ratio: 59M/52F) with degenerative lumbar spinal stenosis-related symptoms such as intermittent claudication [28]. The diagnostic criteria for the stenosis group were based on the combination of symptoms and signs together with the imaging findings (cross-sectional area of the dural sac was less than 100 mm^2^) [29, 30]. The exclusion criteria for this study were individuals under 40 years of age as well as those with congenital stenosis (AP diameter of the bony canal < 12 mm) [31], fractures, spondylolysis, spondylolisthesis, tumors, Paget's disease, steroid treatment, severe lumbar scoliosis (>20 degrees), and iatrogenic conditions.

### 2.2. Computed Tomography (CT) Scans

A high-resolution CT image (Brilliance 64, Philips Medical Systems, Cleveland, OH; slice thickness 0.9–3 mm, voltage 120 kV, current 150–570 mA) was utilized which enabled scan processing in all planes. The images were taken as the participants were in the supine position with extended knees. This study was approved by the Ethics Committee of the Carmel Medical Center (0083-07-CMC).

### 2.3. Facet Joint Orientation (FO)/Facet Angle

**Figure 1:**
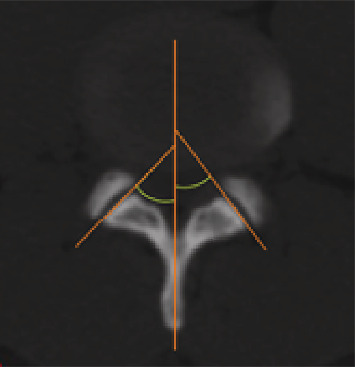
The measurement of facet joint orientation.

The facet joint orientation (FO)/facet angle was measured in the axial plane (bone window) at the level of the intervertebral disc following the method of Noren et al. [22] and defined as the angle between the midsagittal line of the vertebra and the line drawn between the two margins of each of the superior articular facets (Figure 1). The average value of the right and left sides was then calculated.

### 2.4. Facet Tropism (FT)

Facet tropism (FT) was defined as a case in which the absolute number of the difference between the left and right facet orientation angles at each disc level was greater than 7 degrees [25].

### 2.5. Cross-Sectional Area of Dural Sac (CSAD)

The cross-sectional area of dural sac (CSAD) was measured in the axial plane at the lumbar intervertebral disc level [32].

### 2.6. Statistical Analysis

The statistical analyses were done via SPSS version 20 and the parametric variables were checked for distribution. A chi square test and independent *t*-test analysis were used to compare FT, FO, and CSAD between study groups (control and DLSS). A logistic regression analysis via the “Forward LR” method was used (separated by gender) to define the association between DLSS and facet structure/alignment (dependent variable: DLSS; independent variables: FT, FO, age, and BMI). Reliability of the measurements (repeated measurements of 20 individuals) was evaluated using the intraclass correlation (ICC) coefficient test. The first author (JA) took the measurements twice within intervals of 3-5 days in order to assess intratester reliability of the measurements. Intertester reliability involved the first author (JA) and a senior spine surgeon (KH) who took the measurements within an hour of each other. Both testers were blinded to the results of the measurements. Significant difference was set at *P* < 0.05.

## 3. Results

The intratester and intertester reliability results for FO and CSAD were high: 0.971 to 0.933 and 0.930 to 0.890, respectively.

The outcomes of mean age, BMI, and CSAD in both study groups for each gender separately are presented in Table 1.

### 3.1. Facet Orientation and Tropism in the Study Groups

The average of facet angles was significantly smaller in stenosis males (L2-3 to L4-5) and females (L2-3 to L5-S1) compared to their counterparts in the control group (Table 2).

**Figure 2:**
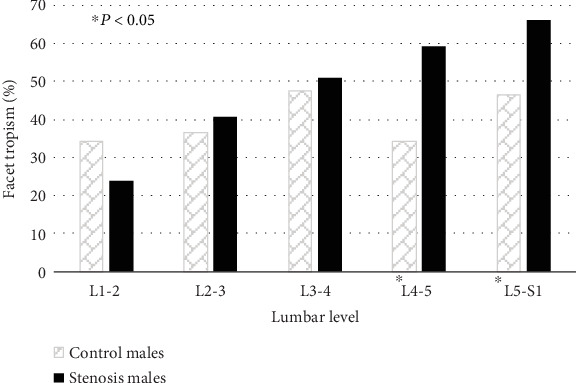
Prevalence (%) of facet tropism in both study groups (control and stenosis), by lumbar level, males only.

**Figure 3:**
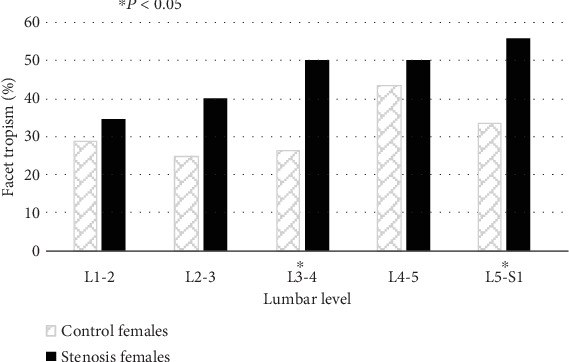
Prevalence (%) of facet tropism in both study groups (control and stenosis), by lumbar level, females only.

The prevalence of FT was significantly greater in stenosis males (L4-5 and L5-S1) and females (L3-4 and L5-S1) compared to their counterparts in the control group (Figures 2 and 3).

Our results also indicate that FO (L2-3, L4-5) and FT (L4-5, L5-S1) in males are significantly associated with DLSS (Table 3). In females, the presence of FT (L3-4 and L5-S1) and sagittal facet angle at L3-4 segment increase the likelihood of DLSS development.

## 4. Discussion

The results of the current study indicate that FT and FO are significantly associated with DLSS. Additionally, facet tropism from L3-4 to L5-S1 increases the risk of developing DLSS by 2.4 to 3.4 times.

It is well-accepted that CT scanning is a superior radiologic modality for the evaluation of both facet joint morphology and degenerative spinal stenosis [7, 33].

Degenerative lumbar spinal stenosis (DLSS) is a common condition in the elderly population, characterized by three-joint complex degeneration [34], ligamentum flavum thickening [35] and osteophytes formation. It has been proposed that the three-joint complex cascade begins at the disc, which loses its height and results in laxity of the facet capsule, leading to increased mobility at the spine segment [36]. Additionally, continuous abnormal segment mobility causes excessive movement of the facets that subsequently leads to cartilage erosion and facet arthritis.

The association between facet orientation and DLSS has been recently mentioned by Liu and colleagues who reported that individuals with DLSS had remarkable sagittal lumbar facet orientation (from L2-3 to L5-S1) compared with the control group [27]. Moreover, one study has shown that patients with sagittally oriented facets have narrow osseous spinal canals [37]. Grobler and colleagues previously reported that subjects with DS display greater sagittal orientation of L4-5 facet joints compared to both normal population and spinal stenosis patients without DS [38]. However, no significant difference was found between stenosis subjects and the normal population.

We found no previous study that has addressed the association between FT and DLSS except for the relationship with DS, hence the lack of comparative analysis.

The association between sagittal FO and DLSS is not surprising since there are sufficient evidences indicating that sagittal orientation of the facets augments the shear forces in the spine segment leading to facet arthrosis and degenerative spondylolisthesis [39, 40]. Previous studies have also reported that the prevalence of facet arthrosis and DS is greater in the DLSS population compared to the general population [41].

The lumbar spine unit function depends on the congruity of the intervertebral disc anteriorly and two facet joints posteriorly. The facet joint has an essential role to resist forward displacement and rotation [42] and carries about 16% of the load on the lumbar spine [43].

Our study demonstrated that both FO and FT are considered risk factors for the development of symptomatic DLSS. This result implies that (a) FT and FO could be two different entities and (b) the trigger for spine segment degeneration could begin at the posterior elements such as the facet joints rather than the intervertebral disc. Our results can be supported by the study of Miyasaki et al. that proposed that patients with sagittally oriented facets might be predisposed to lumbar spinal canal stenosis [37].

A systematic review and meta-analysis study has recently confirmed that individuals with DS at L4-5 have more remarkable FT and sagittal FO [10]. Although it is not apparent whether FO and FT act as primary manifestation or secondary reconstruction [44, 45], our results lend support to the notion that FT could accelerate both disc disease and DS. Some studies have reported that FT has the potential to alter the biomechanical nature of the lumbar spine causing pathologic changes in the intervertebral disc [6, 46]. Boden and colleague, for example, have reported that the sagittally oriented facet joints at L4-L5 were significantly associated with disc herniation. It has also been reported that FT makes the corresponding segment more vulnerable to external moments or anterior shear force [47]. Cyron and Hutton suggested a relationship between FT and instability [2]. Furthermore, one study has reported that subjects with disc herniation exhibited asymmetry and sagittalization of facet joints [48]. Park and colleagues have mentioned that the degree of FT may influence the type of disc herniation (e.g., far lateral or posterolateral) [49].

It is noteworthy that only the lower lumbar facets (L3-4 to L5-S1) contribute to DLSS development, as these levels are more susceptible to aging and degeneration [6, 11, 15].

Finally, it is interesting to mention that, in males, FT at L4-5 and L5-S1 levels are related to DLSS, whereas, in females, the corresponding facets are at L3-4 and L5-S1. This discrepancy between males and females can be partially explained by the difference in the lumbar lordosis shape as Hay et al. have reported that females have greater curvedness than males [50].

In summary, we postulate that FT and sagittal FO are two different entities that could lead to disc degeneration and facet arthrosis, which in some cases (e.g., combined with greater BMI) may harm the spine stability thus leading to lumbar spinal stenosis.

### 4.1. Limitation of the Study

This is a retrospective study, and the outcomes should be supported by well-established prospective studies. Additionally, we examined facet angles in an axial plane; however, facet structures are complicated 3-dimensional structures, and this fact should be considered in further studies.

## 5. Conclusions

Our results indicate that both FT and facet sagittal orientation in the lower lumbar spine are risk factors for DLSS development. However, no causal association is mentioned.

## Figures and Tables

**Table 1:** Age, BMI, and CSAD (L1-2 to L5-S1) of the study groups (control vs. stenosis) for each gender separately.

Variables	Males			Females		
	Control (mean ± SD)	Stenosis (mean ± SD)	*P* value	Control (mean ± SD)	Stenosis (mean ± SD)	*P* value
Age (years)	62.1 ± 12	64.6 ± 10	0.214	60.4 ± 12	62 ± 9	0.429
BMI (kg/m^2^)	27.3 ± 4	29.3 ± 4	0.009	27.7 ± 5	30.8 ± 5	0.002
CSAD L1-2 (mm^2^)	191 ± 41	124 ± 47	<0.001	199 ± 39	143 ± 33	<0.001
CSAD L2-3 (mm^2^)	162 ± 40	81 ± 37	<0.001	171 ± 44	103 ± 38	<0.001
CSAD L3-4 (mm^2^)	148 ± 40	60 ± 23	<0.001	150 ± 42	67 ± 31	<0.001
CSAD L4-5 (mm^2^)	148 ± 51	53 ± 31	<0.001	146 ± 47	47 ± 20	<0.001
CSAD L5-S1 (mm^2^)	157 ± 55	101 ± 44	<0.001	142 ± 49	83 ± 32	<0.001

BMI: body mass index; CSAD: cross-sectional area of dural sac; SD: standard deviation.

**Table 2:** Facet orientation (FO) values of the study groups (control vs. stenosis) by lumbar levels, for each gender separately.

Variables	Males			Females		
	Control (mean ± SD)	Stenosis (mean ± SD)	*P* value	Control (mean ± SD)	Stenosis (mean ± SD)	*P* value
FO L1-2 (degree)	23.6 ± 8	21.4 ± 9	0.133	24.4 ± 7	21.7 ± 10	0.079
FO L2-3 (degree)	26.6 ± 8	21.2 ± 8	<0.001	27.6 ± 6	21.9 ± 9	<0.001
FO L3-4 (degree)	34 ± 10	27.8 ± 9	<0.001	36 ± 7	26.6 ± 10	<0.001
FO L4-5 (degree)	43 ± 9	35.6 ± 11	<0.001	45.8 ± 8	37.7 ± 11	<0.001
FO L5-S1 (degree)	49.8 ± 9	46.9 ± 9	0.073	52.2 ± 8	46.2 ± 8	<0.001

SD: standard deviation.

**Table 3:** A logistic regression analysis demonstrating the variables that are significantly associated with degenerative lumbar stenosis (males and females listed separately).

	OR	CI 95%	*P* value
Males			
BMI	1.095	1.001-1.198	0.048
FO L2-3	0.944	0.898-0.993	0.024
FO L4-5	0.948	0.908-0.988	0.013
FT L4-5	3.389	1.507-7.621	0.003
FT L5-S1	2.436	1.078-5.507	0.032
Females			
BMI	1.144	1.050-1.245	0.002
FO L3-4	0.877	0.831-0.925	<0.001
FT L3-4	3.195	1.266-8.060	0.014
FT L5-S1	2.962	1.176-7.460	0.021

OR: odds ratios; CI: confidence intervals; BMI: body mass index; FO: facet orientation; FT: facet tropism.

## Data Availability

The data used to support the findings of this study are available from the corresponding author upon request.
